# Hepatitis C virus sequence divergence preserves p7 viroporin structural and dynamic features

**DOI:** 10.1038/s41598-019-44413-x

**Published:** 2019-06-10

**Authors:** Benjamin P. Oestringer, Juan H. Bolivar, Jolyon K. Claridge, Latifah Almanea, Chris Chipot, François Dehez, Nicole Holzmann, Jason R. Schnell, Nicole Zitzmann

**Affiliations:** 1 Oxford Glycobiology Institute, Department of Biochemistry, University of Oxford, South Parks Road, Oxford, OX1 3QU United Kingdom; 2 Department of Biochemistry, University of Oxford, South Parks Road, Oxford, OX1 3QU United Kingdom; 3 Laboratoire International Associé CNRS-University of Illinois at Urbana Champaign, Université de Lorraine, BP 70239, 54506 Vandœuvre-lès-Nancy, France; 4 Department of Physics, University of Illinois at Urbana-Champaign, 1110 West Green Street, Urbana, Illinois 61801 United States; 5 Present Address: Immunocore Limited, 101 Park Drive, Milton Park, Abingdon, Oxon OX14 4RY United Kingdom; 6 Structural Biology Brussels, Vrije Universiteit Brussel, Pleinlaan 2, 1050 Brussels, Belgium; 7 Structural and Molecular Microbiology, Structural Biology Research Center, VIB, Pleinlaan 2, 1050 Brussels, Belgium

**Keywords:** Solution-state NMR, Molecular modelling

## Abstract

The hepatitis C virus (HCV) viroporin p7 oligomerizes to form ion channels, which are required for the assembly and secretion of infectious viruses. The 63-amino acid p7 monomer has two putative transmembrane domains connected by a cytosolic loop, and has both N- and C- termini exposed to the endoplasmic reticulum (ER) lumen. NMR studies have indicated differences between p7 structures of distantly related HCV genotypes. A critical question is whether these differences arise from the high sequence variation between the different isolates and if so, how the divergent structures can support similar biological functions. Here, we present a side-by-side characterization of p7 derived from genotype 1b (isolate J4) in the detergent 6-cyclohexyl-1-hexylphosphocholine (Cyclofos-6) and p7 derived from genotype 5a (isolate EUH1480) in *n*-dodecylphosphocholine (DPC). The 5a isolate p7 in conditions previously associated with a disputed oligomeric form exhibits secondary structure, dynamics, and solvent accessibility broadly like those of the monomeric 1b isolate p7. The largest differences occur at the start of the second transmembrane domain, which is destabilized in the 5a isolate. The results show a broad consensus among the p7 variants that have been studied under a range of different conditions and indicate that distantly related HCVs preserve key features of structure and dynamics.

## Introduction

Approximately 3% of the world’s population carries the hepatitis C virus (HCV), putting more than 200 million people at risk of developing severe liver diseases^1–3^. HCV displays high genetic heterogeneity and is classified into eight genotypes (gt 1–8) and more than a hundred subtypes^4–8^. The polyprotein precursor is expressed from a 9.6 kb positive-sense, single-stranded RNA genome ((+) ssRNA)) and is co- and post-translationally cleaved by cellular and viral proteases to produce at least ten viral proteins^9–11^. In the HCV polyprotein precursor, p7 lies between the structural and non-structural proteins. It is essential for the assembly and secretion of infectious viral particles *in vitro*^12–16^ and for virus propagation *in vivo*^17^, making it an attractive therapeutic target^18^.

HCV p7 is a small, hydrophobic protein comprising 63 amino acids^19^. The structural properties of p7 constructs derived from 1b genotypes have been widely investigated using a range of approaches^18^, including electron microscopy (EM)^20–22^, nuclear magnetic resonance spectroscopy (NMR)^23–31^, and molecular modeling^22,24,32–36^. The various NMR studies of monomeric p7 suggest a similar architecture that is in broad agreement with secondary structure prediction from its amino-acid sequence, namely two hydrophobic transmembrane (TM) regions separated by a conserved basic loop region^9,19^. Analytical ultracentrifugation measurements suggest that p7 self-assembly results predominantly in hexameric and heptameric viroporins, shown in electrophysiology experiments to be cation selective^23^. Computational modeling studies of p7 predict that the monomeric protein adopts hairpin-like structures, which then associate side-by-side with the N-terminal TM helix forming the channel pore^22,24,32,34–36^.

**Figure 1:**
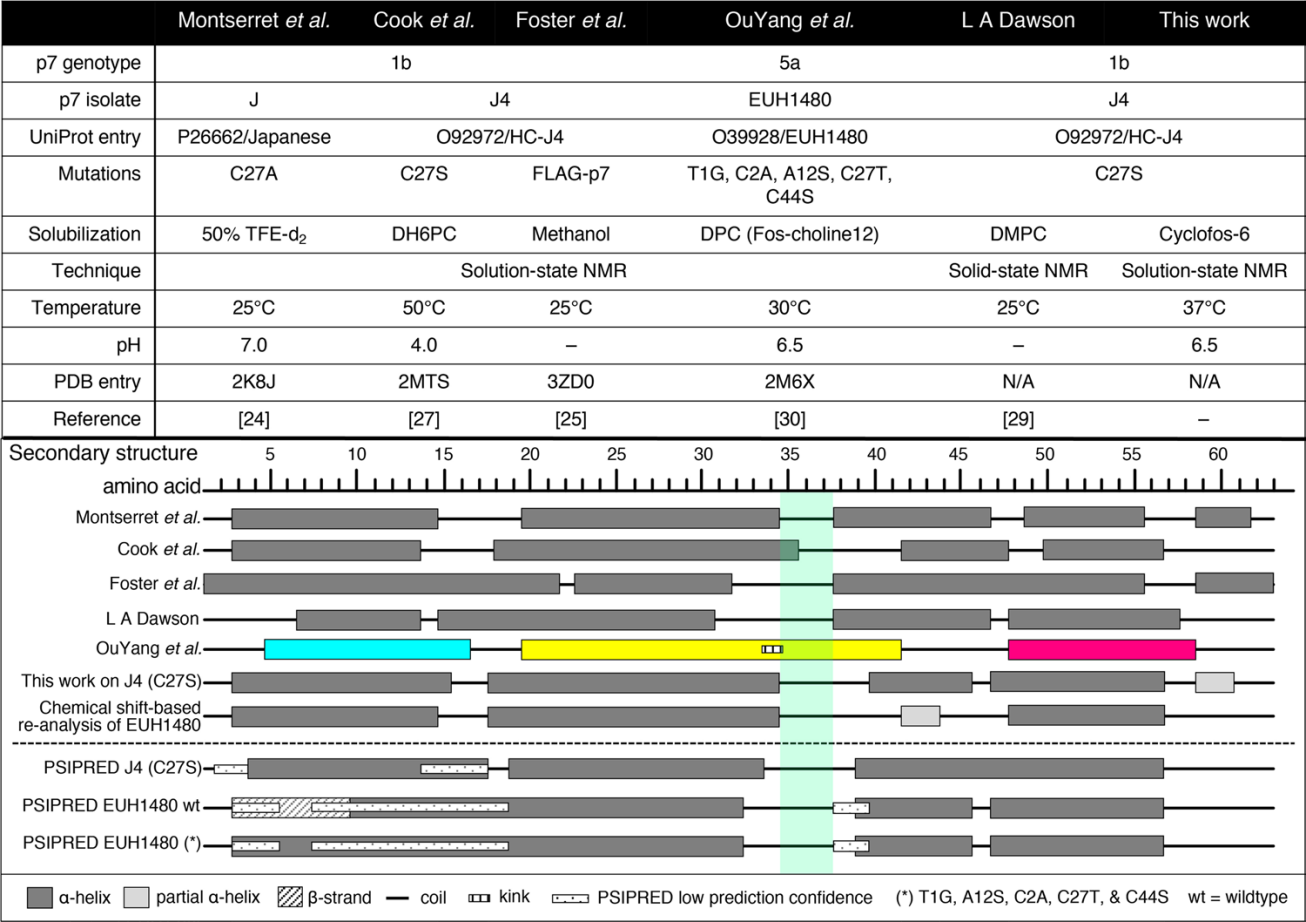
Comparison of p7 secondary structures. Sample conditions (top) and secondary structure determinations (bottom) reported for p7 monomers and the hexamer of OuYang *et al*. The sample conditions used for p7(5a/EUH1480) in this work are the same as previously reported^29^. For comparison, the PSIPRED secondary structure predictions based on amino acid sequences for p7(1b/J4) and both wildtype and mutated (*) p7(5a/EUH1480) are also shown^82^. The PSIPRED prediction for J4 with the C27S substitution is identical to wildtype J4 (not shown). For the subunit structure reported by OuYang *et al*., the helices are color-coded according to the original paper^29^. The basic loop region ~33–37, which differs notably between monomeric genotype 1b p7 and oligomeric 5a p7, is indicated by green shading^23,24,26,28,29^. The secondary structures were estimated directly from published data (secondary chemical shifts, NOEs, and/or dipolar waves). In the case of p7(5a/EUH1480)^29^, the helical regions were determined from the structure deposited in the PDB (2M6X). Plotted helical regions by residue number: Montserret *et al*. 2010: 3–14, 20–34, 38–46, 48–55 and 59–61; Cook *et al*. 2013: 3–13, 18–35, 42–47 and 50–56; Foster *et al*. 2014: 1–21, 23–31, 38–55 and 59–63; LA Dawson: 7–13, 15–30, 38–46 and 48–57; Ouyang *et al*. 2013: 5–16, 20–41 and 48–58; Oestringer *et al*. 2018 J4: 3–15, 18–34, 40–45, 47–56 and 59–60; Chemical shift-based re-analysis of EUH1480 using published^29^ chemical shifts: 3–14, 18–34, 42–43 and 48–56; PSIPRED J4: 4–17, 19–33 and 39–56; PSIPRED EUH1480: 3–32, 39–45 and 47–56; PSIPRED EUH1480 mt5: 3–9 (beta strand), 10–32, 39–45 and 47–56.

A strikingly different subunit conformation and packing arrangement was reported for a p7 construct based on a 5a genotype^29^. Several features of the hexameric structure determined by NMR were unexpected based on previous structural and functional studies of p7, but recent experiments indicate that the protein is not oligomeric in the detergent *n*-dodecylphosphocholine (DPC) used to derive the structure^37^. The secondary structure of the genotype 5a construct, which should be unaffected by the introduction of tertiary restraints in the structure determination, also indicated key differences compared to monomeric structures published hitherto (Fig. 1 and references therein).

HCV has one of the highest mutation rates and genetic variability amongst RNA viruses^5,38^, and the unique genotype 5a p7 construct used to determine an oligomeric structure is very different from the genotype 1b constructs used to derive all other structures (Supplementary Fig. 1). Whereas the function of several genotype 1 and 2 constructs have been studied^20,23,39–45^, attempts at recording electrophysiological measurements of genotype 5a p7 were not successful, making it unclear how this p7 relates to the more commonly studied homologs^29^.

We sought to understand the extent to which sequence divergence has resulted in differences in structural preferences for p7. The structure, dynamics, and solution properties of p7 from genotype 1b (isolate J4) were studied alongside that of genotype 5a (isolate EUH1480) in phosphocholine-based detergents, using a combination of solution NMR spectroscopy and molecular dynamics (MD). The largest differences were detected in residues 40–45, which appear to adopt an unstable helical structure that may be sensitive to solution conditions. The two constructs were otherwise very similar in secondary structure and dynamics, indicating that key features of p7 structure and dynamics are conserved between distantly related HCV.

## Results

### Sample preparation and spectral characterization

HCV p7 from genotype 1b isolate J4 containing a C27S mutation to prevent disulfide bond formation during preparation^25–27^ (referred to as p7(1b/J4)) was expressed and purified from *E*. *coli* and solubilized directly into the detergent 6-cyclohexyl-1-hexylphosphocholine (Cyclofos-6) enabling solution NMR analyses of the protein at pH 6.5 and 37 °C. The spectral quality was unaffected by changes in pH between 6 and 7 or detergent concentrations between 26.8 mM and 268 mM, indicating no change in the structure or oligomeric state over these ranges.

**Figure 2:**
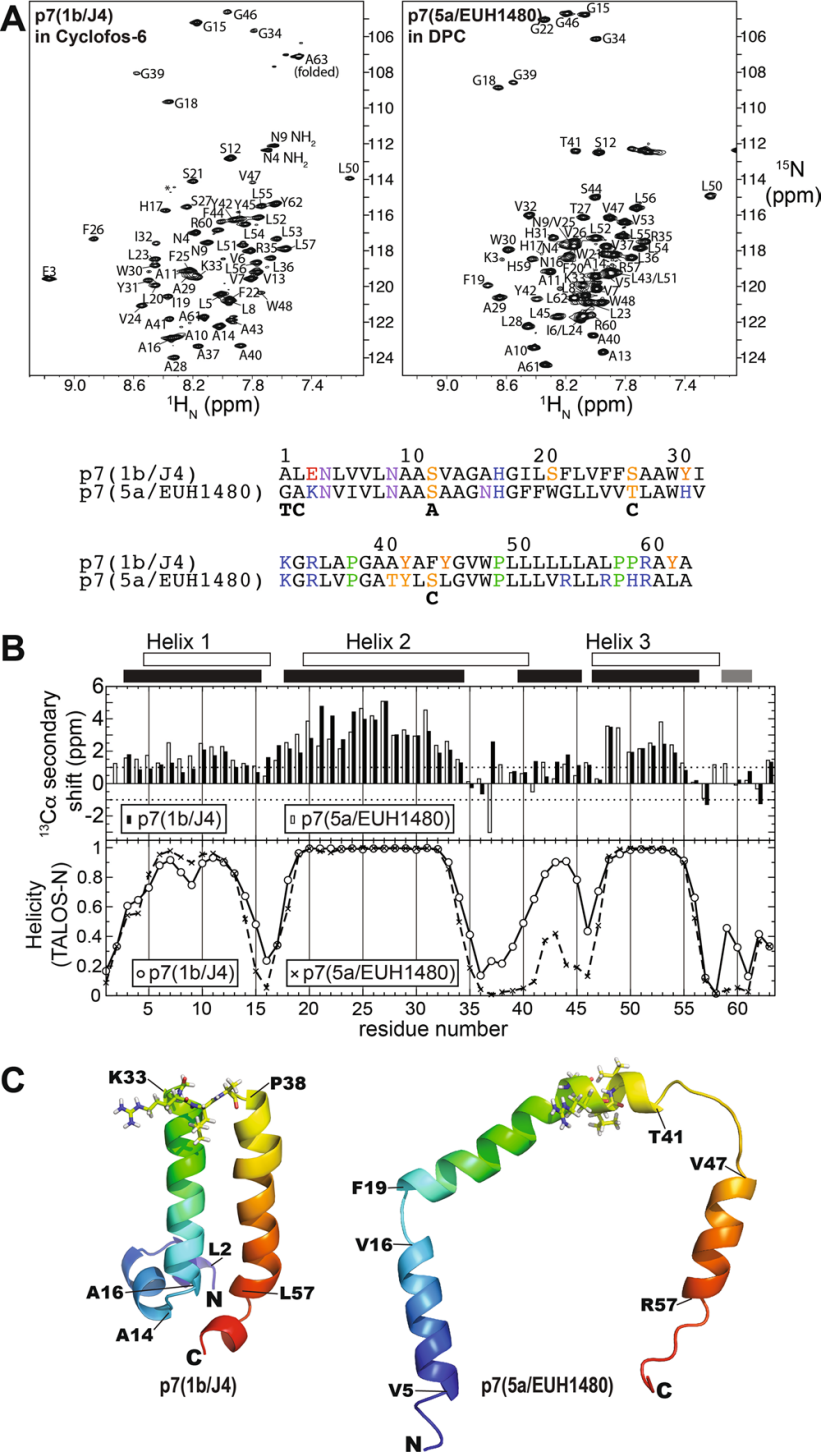
Spectral characterization of HCV p7 isolates. (**A**) 2D ^1^H-^15^N HSQC spectra of p7(1b/J4) in Cyclofos-6 (left), and p7(5a/EUH1480) in DPC (right), with amino acid assignments indicated. The amino acid assignments of the p7(5a/EUH1480) spectrum were transferred from^29^. The amino acid sequences of the constructs are shown below the spectra. Below the sequence for p7(5a/EUH1480) are indicated the native amino acids that were substituted in this construct used for experiments here and in^29^. (**B**) Top: ^13^Cα secondary chemical shift index for p7(1b/J4) (filled bars) and p7(5a/EUH1480) (open bars), with positive values indicative of α-helical conformation. Bottom: TALOS-N^46^ chemical shift-based secondary structure prediction for p7(1b/J4)(⚪) and p7(5a/EUH1480) (X). Chemical shift data for p7(5a/EUH1480) were taken from the BioMagResBank entry 19162^29^. Shown above the plot and indicated by unfilled and filled bars are the helical residues determined for p7(5a/EUH1480) from PDB 2M6X and those predicted from chemical shifts for p7(1b/J4), respectively. (**C**) Left: Membrane CS-Rosetta-based structure of p7(1b/J4). Right: A single subunit of p7(5a/EUH1480) showing the horseshoe-like conformation in the oligomeric model 2M6X^29^ is shown for comparison. The structures are shaded from blue (N-terminus) to red (C-terminus) and the residues 34–37 are shown as sticks. The residues at the beginning and end of each helix are indicated.

The p7 construct based on genotype 5a isolate EUH1480 was expressed in *E*. *coli*, purified, and refolded into the detergent DPC as described in reference^29^. The 5a construct contained five substitutions (T1G, C2A, A12S, C27T, and C44S) to enable a direct comparison with a previously published structure^29^ and is referred to as p7(5a/EUH1480). The resulting ^1^H,^15^N-based spectra collected on p7(5a/EUH1480) were similar to those previously reported, confirming that the protein conformation and environment were similar to those samples used previously^29^ and enabling analyses of additional NMR experiments based on published chemical shift values. High quality spectra could be recorded on both p7(1b/J4) and p7(5a/EUH1480) at 37 °C using conventional, non-TROSY approaches on fully protonated protein (Fig. 2A), consistent with previously published SEC-MALS experiments indicating that the protein is monomeric under these conditions^37^. The spectral properties of p7(1b/J4) were similar to those of p7(5a/EUH1480) and the backbone resonances (^1^H_N_, ^15^N, ^13^Cα, ^13^C′) of p7(1b/J4) were assigned using conventional triple-resonance experiments.

Secondary structure probabilities were determined from chemical shifts of p7(1b/J4) and p7(5a/EUH1480), with chemical shift data for p7(5a/EUH1480) taken from the BioMagResBank (entry 19162)^29^. The chemical shift-based predictions from TALOS-N^46^ were similar for the two constructs in helix 1 (residues ~3–15), helix 2 (~18–34), and the C-terminal half of helix 3 (residues ~48–56). In addition to helical breaks centered at residues 16 and 35–37, a discontinuity in helix 3 was observed in both constructs at G46, which is three residues before a proline (P49). Differences between the two constructs were apparent in the N-terminal residues of helix 3 (residues ~40–45) in p7(5a/EUH1480), which were significantly destabilized compared with p7(1b/J4) (Fig. 2B). In addition, a weak propensity for a C-terminal helix beginning at residue P59 was observed for p7(1b/J4). Surprisingly, the helical residues present in the subunits of the hexameric model of p7(5a/EUH1480) from OuYang *et al*. (PDB 2M6X)^29^ are markedly different from the secondary structure derived here from their chemical shift data (Fig. 2B).

A structural model of p7(1b/J4) was generated using chemical shift Rosetta (CS-Rosetta)^47^ with a membrane force field^48^. The structure, shown in Fig. 2C (left), is most similar to the structures determined previously by Cook *et al*.^26^, using a similar approach, and by Montserret *et al*.^23^. In the chemical shift-based Rosetta structure of p7(1b/J4), residues 16–33 (helix 2) correspond to the first TM domain, residues 34–37 form a loop, and residues 38–57 (helix 3) correspond to the second TM domain. By contrast, in the subunit structure taken from the hexameric model of p7(5a/EUH1480)^29^ (shown at right in Fig. 2C) residues 34–37 are helical and residues ~42–46 form a loop.

### Amide backbone ^15^N relaxation and proton exchange

**Figure 3:**
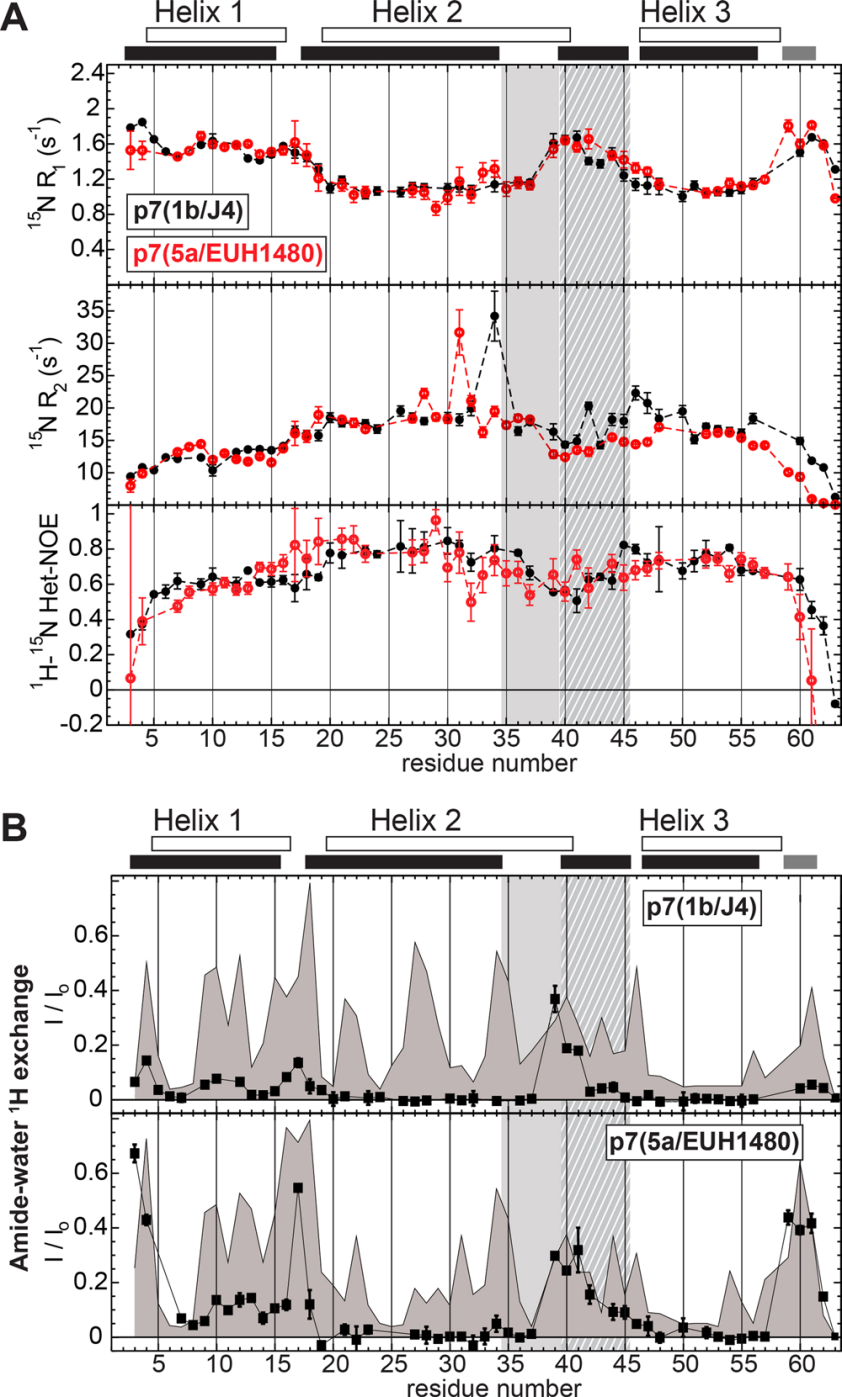
HCV p7 backbone amide dynamics and amide-water proton exchange. (**A**) ^15^N R_1_, ^15^N R_2_, and ^1^H-^15^N heteronuclear NOEs as a function of residue number for p7(1b/J4) (filled black circles) and p7(5a/EUH1480) (open red circles). The backbone amide heteronuclear NOE value for residue A63 of p7(5a/EUH1480) was −2.1 (data point not shown). (**B**) Backbone amide hydrogen exchange data for p7(1b/J4) (top) and p7(5a/EUH1480) (bottom) as a function of residue number. NMR CLEAN chemical exchange (CLEANEX) experiments^83^ were recorded using a 50 ms (◼) mixing time. For comparison are shown the empirically-based predictions of the magnitude of amide exchange from primary structure alone (solid line with grey shading)^84^. Experiments for (**A**,**B**) were recorded at 600 MHz (^1^H) and 37 °C. The relaxation data were collected using conventional HSQC-based pulse sequence experiments. Shown above the plots in (**A**,**B**) by unfilled and filled bars are the helical regions determined for p7(5a/EUH1480) from PDB 2M6X and those predicted from chemical shifts for p7(1b/J4), respectively. The light shading for residues 35–39 indicate helical residues in the 2M6X structural model that were found in this study to be nonhelical. The crosshatching for residues 40–45 indicate residues predicted to form an unstable helix.

Backbone dynamics of p7(1b/J4) and p7(5a/EUH1480) were characterized by ^15^N R_1_ and R_2_ relaxation rates and {^1^H-^15^N} heteronuclear Overhauser effects (hetNOEs). Trimmed mean averages of the backbone amide ^15^N R_1_ and R_2_ values were used to calculate apparent molecular weights of the protein and detergent complex (see Experimental Methods). The derived rotational correlation times, τ_c_, were 10.6 ns and 10.1 ns at 37 °C for p7(1b/J4) and p7(5a/EUH1480), respectively. These values correspond to apparent molecular weights of ~40 kDa, and are consistent with monomeric p7 embedded in micelles of ~30 kDa and consistent with SEC-MALS studies of p7(5a/EUH1480)^37^. Elevated R_1_ values, and depressed R_2_ and hetNOE values, are indicative of increased flexibility on the picosecond-nanosecond (ps-ns) timescale, as can be seen for the N- and C-termini (Fig. 3A). For both constructs, increased ps-ns timescale flexibility was apparent in helix 1, whereas the least flexible regions, as indicated by lower R_1_ values and higher R_2_ and hetNOE values, were in the middle of helices 2 and 3. Increased ps-ns flexibility is observed for both constructs in loop residues G39, A40, and A41 (T41 in p7(5a/EUH1480)). The relatively high R_2_ values observed for G34 of p7(1b/J4) and H31 of p7(5a/EUH1480) indicate local dynamics on the microsecond-millisecond timescale. The nonhelical residues 35–37 exhibited little or no increase in dynamics compared with the TM domain residues of helix 2 indicating they form a structured loop.

While the per-residue profiles for backbone dynamics of p7(1b/J4) and p7(5a/EUH1480) were broadly similar, differences were seen in residues 39–47 (Fig. 3A). These residues are typically assigned to the start of the second TM domain (Fig. 1) but exhibited low helical propensities in both constructs (Fig. 2B), and increased ^15^N R_1_ and decreased ^15^N R_2_ in p7(5a/EUH1480) indicate that these residues in p7(5a/EUH1480) are significantly more dynamic than those in p7(1b/J4).

The rates of backbone amide proton exchange with water provide additional insights into secondary structure and dynamics since the proton exchange rates reflect hydrogen bond participation in addition to sidechain-dependent intrinsic exchange rates and solvent accessibility. Similar patterns of exchange were observed for the two constructs (Fig. 3B). In particular, H17, which is in a break between helices 1 and 2 exhibited elevated exchange rates, as well as residues 39–41. As expected, amides in the N- and C-termini also showed increased proton exchange rates for both constructs, but the magnitudes in p7(5a/EUH1480) were much higher than that of p7(1b/J4) and approached values expected for the same sequence fully unstructured in water^49,50^ (Fig. 3B). Several residues in helix 1 of both constructs also exhibit amide proton exchange consistent with this helix being solvent exposed or only transiently bound to the micelle surface.

### MD-simulation of the p7(1b/J4) structural model in POPC bilayers

**Figure 4:**
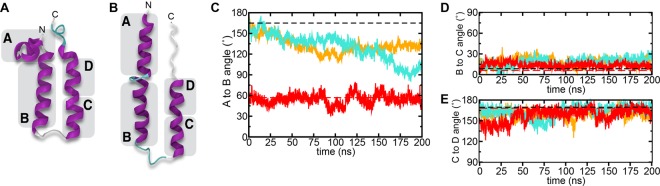
MD simulations of p7 structures. Structural evolution of p7 hairpin monomer structures in POPC bilayers measured over 200 ns MD trajectories by means of the angles formed by contiguous α-helical segments for (**A**) the p7(1b/J4) Rosetta-derived hairpin structure, and (**B**) the hairpin structure of Montserret *et al*. (architecture used for both p7(1b/J) and p7(5a/EUH1480) sequences). (**C**–**E**) Show the time traces of the angles between the helices for p7(1b/J4) (this work; red), p7(1b/J) from Montserret *et al*. (orange), and the sequence of p7(5a/EUH1480) made to adopt the hairpin conformation of Montserret *et al*. (cyan)^51^. Horizontal dashed lines correspond to the starting angles of structures based on that of Montserret *et al*.^23^ (dashed black) or of p7(1b/J4) from this study (dashed red).

Previous atomistic MD simulations have indicated that monomeric forms of the p7(5a/EUH1480) sequence and the p7 sequence from genotype 1b isolate J (p7(1b/J)) could form stable hairpins in 1-palmitoyl-2-oleoylphosphatidylcholine (POPC) bilayers over a 200 ns timescale^51^. Identical simulations were carried out for the p7(1b/J4) Rosetta-derived structure to examine whether the hairpin conformation of p7(1b/J4) is also stable in a POPC bilayer. The results are compared with those from p7(1b/J) and p7(5a/EUH1480)^51^ in Fig. 4. Visual inspection of snapshots from the simulation did not reveal any large-scale alteration of the structures over the timescale of the simulation. To quantitatively analyze the conformational evolution of the monomeric protein, the structures were separated into four α-helical segments conserved throughout the simulations as observed previously^51^. Helix A (residues 4–14) corresponds approximately to helix 1 identified by secondary chemical shifts, helix B (20–31) corresponds to ~helix 2, and helix C (39–47) and D (48–54) correspond to ~helix 3 broken by the kink around G46 (Fig. 4). Over the 200 ns simulation the maximum variation in the angles between helices B and C, and between C and D were ~30° and ~40°, respectively, indicating that the hairpin conformations are stable over the duration of the simulations. By contrast, the variations in the angle between helices A and B were larger, and up to 80° for p7(5a/EUH1480). Variation in the angle between helices A and B arises from movements of helix A, which was not embedded in the hydrophobic portion of the membrane in the models of p7(1b/J) and p7(5a/EUH1480). Helix A in the Rosetta-derived structure of p7(1b/J4) adopts a membrane surface-bound conformation that is more stable over the duration of the simulation than the detached conformation of p7(1b/J) and p7(5a/EUH1480). The increased flexibility and solvent accessibility observed by NMR for this helix (Fig. 3) is consistent with it being outside of the micelle or loosely associated with the micelle surface.

## Discussion

HCV p7 is a viroporin essential for infectious virus production and therefore a potential drug target^52,53^, with the search for a truly ‘pan-genotypic’ HCV treatment ongoing. Current standard treatment of care using the nucleotide analog sofosbuvir targeting the HCV protein NS5B is genotype-dependent in its ability to overcome innate viral resistances and generally requires combinations with other direct acting antivirals to prevent escape mutations^54,55^, hence several compounds targeting p7 have been investigated and could serve as anti-HCV drugs. Some of these compounds are postulated to inhibit the assembled channel whereas others are hypothesized to disassemble the oligomeric channel^56^ (reviewed in references^18,53^). Thus, detailed knowledge of the p7 monomer structure and how it assembles to form functional channels remains of great interest. However, p7 poses a challenge for structural studies because most of the protein is buried in the membrane. In addition, it forms oligomers with variable stoichiometry and may only weakly oligomerize in detergent^20–23,34^. Thus despite its small size only one atomic-resolution, experimental model for an assembled p7 channel has been published^29^. However, that structure included several unexpected features which could not be reconciled with many published structural, biochemical, and functional data and it was subsequently shown that the detergent used to solubilize the protein for those structural studies does not support p7 oligomerization^37^. Further questions remain about the secondary structure in that p7 model, which is not expected to be strongly affected by introducing tertiary contacts in the structure calculations. While phosphocholine detergents are strongly denaturing and can introduce artefacts^57^, the persistence of structural features across different solubilizing reagents, solution conditions, and sequences can provide an indication of whether those features are likely to be present in a biological membrane.

The structures of two closely related isolates from 1b (p7 from isolate J and J4; 94% identical) have been studied previously (Fig. 1). For these 1b-derived p7 constructs studied under different conditions, the largest differences were seen for an N-terminally FLAG-tagged construct in methanol that has a long N-terminal helix extending from the FLAG-tag to residue ~21 and lacks the break at position ~15 seen in other studies. By contrast, the 1b- and 5a- derived p7 sequences are two of the most distantly related among known p7 sequences (Supplementary Fig. 1). The sequences of the p7(1b/J4) and p7(5a/EUH1480) constructs studied here differ in 46% (29 of 63) of the positions, providing a good test of whether structural features of p7 are conserved.

The structure in the region around the strictly conserved dibasic motif (residues K/R33 and K/R35) is of particular interest from a functional point of view since charge neutralizing substitutions result in nonviable virus^17^. The dibasic motif is known to be important in ion channel activity^58^, and double glutamine or double alanine substitutions in the JFH-1 isolate of genotype 2a result in 100- and 1,000-fold decreases in total infectivity, respectively^16^. The oligomer model of the p7(5a/EUH1480) construct indicated that helix 2 extends to residue T41 with a kink of ~45° at G34, whereas the results here indicate that helix 2 ends at ~G34 in both p7(1b/J4) and p7(5a/EUH1480). Comparison with previously published work on monomeric p7 indicates a consensus that the cytosolic loop is in residues 35–37 (Fig. 1), such that R35 is within the loop and K33 is close enough to the end of the TM domain to “snorkel” into the lipid headgroup region^59^.

The largest structural and dynamic differences between the two distantly related constructs studied here are in residues ~40–45, which are immediately C-terminal to the cytosolic loop. The differences seem to arise from increased sequence variability and structural instability in this region. The second TM domain of p7 is typically assigned to residues ~40–56, with a discontinuity at 46 or 47. Residues 48–56 exhibit strong helicity in most studies, whereas residues 40–45 tend to be more variable. Structural instability was observed previously in this region for the p7(1b/J4) construct in different conditions^26^. In the work reported here, residues 40–45 exhibit moderate to high helicity in p7(1b/J4) but are mostly nonhelical in p7(5a/EUH1480). Sequence conservation in residues 40–45 is generally low (Supplementary Fig. 2), and only two of the positions in this region are conserved between 1b/J4 and 5a/EUH1480. Structural variation is also seen here between the 1b-derived p7 constructs having no or very small sequence differences but studied in different membrane mimetics (Fig. 1). The composition of residues 40–45 tends to be relatively hydrophilic for a TM domain (Supplementary Fig. 3), which can make it particularly sensitive to the membrane mimetic, and the helix here may be more stable in lipid membranes^28^.

A helix discontinuity at, or near, G46 was seen in both constructs and also observed in other studies^23,24,26^. The discontinuity is likely due in part to the proline at position 49. Prolines are often C-terminal to helical kinks and are present at higher frequency within TM helices than in helices of water-soluble domains^60^. Kinks can be exaggerated by the detergent environment^61^, but MD simulations in lipid membranes are also consistent with at least a small (~15–30°) deflection of the helix (Fig. 4E). Although G46 and P49 are not strictly conserved, one or the other is always present and substitutions in these positions are for more hydrophilic amino acids (Supplementary Fig. 2).

The published subunit structure of p7(5a/EUH1480) pointed to a helix extending from 20–41 with a kink at G34^29^, whereas secondary chemical shifts indicate that residues 35–41 are not helical, and 39–41 are particularly flexible (reference^62^ and Fig. 2B). The helical boundaries determined for the subunit structure were based on observation of characteristic NOEs, which is expected to yield similar results compared with chemical shift-based approaches. However, spectral overlap in the ^1^Hα region can result in information gaps with the NOE approach and chemical shift information tends to be more complete since ^13^C shift information (^13^C′, ^13^Cα, ^13^Cβ) is partially redundant. Consistent with a non-helical state, these amides exhibit proton exchange rates comparable to what would be expected for the same sequence unstructured in water (Fig. 3B).

The pattern of amide proton exchange of p7(5a/EUH1480) in DPC was similar to that of p7(1b/J4) in Cyclofos-6, and the results are consistent with the exchange rates previously reported for p7(5a/EUH1480) in similar conditions^30^. Some regions, including the N- and C-termini, helix 1, and H17, exhibited higher exchange rates in p7(5a/EUH1480), which may be due to differences in hydrophobicity between the constructs: The regions including residues 13–20 and 53–58 are more hydrophobic in p7(1b/J4) than p7(5a/EUH1480) (Supplementary Fig. 3), which may result in remodeling of the detergent micelle and more water exposure. Although all substitutions introduced to create p7(5a/EUH1480) decrease its hydrophobicity, the wildtype sequence is also less hydrophobic than p7(1b/J4) in the regions with large differences in amide proton exchange rates. The increased exchange rates and high flexibility in helix 1 (residues ~5–15) that have been attributed either to exposure to a hydrophilic channel pore in the oligomer^30^ or to membrane thinning^62^ may be explained more simply by the hairpin models showing that helix 2 is the TM helix, and helix 1 extends out of the membrane^23,24^ or possibly lies on the membrane surface^26^.

Finally, we provide further evidence that the hairpin conformation can be stably adopted by different p7 sequences. Most p7 modelling studies predict the monomer to adopt a closely packed helical hairpin conformation, which then assembles side-by-side into oligomers. Most studies have used p7 constructs from genotypes 1 or 2, but MD simulations have shown that p7(5a/EUH1480) can also adopt a hairpin conformation in membranes^63^, and that the stability is not strongly sequence dependent^51^. Here, we expand those results to show that the Rosetta-derived hairpin conformation of p7(1b/J4) is also stable in a lipid bilayer.

In conclusion, we find that the genetically distant genotype 1b and 5a p7 constructs behave similarly, as assessed by NMR spectroscopy in detergent and computer modelling in lipid membranes. The results for 5a p7 are largely consistent with previous reports on monomeric p7’s from other genotypes and in a range of solubilizing conditions, demonstrating that key structural and dynamic features are conserved among distantly related p7 isolates. Together, these findings provide a foundation for future studies of how p7 monomers assemble to form an oligomeric viroporin.

## Experimental Section

### Protein expression and purification

HCV p7 (strain J4, C27S, genotype 1b, “p7(1b/J4)”, and strain EUH1480, T1G/C2A/A12S/C27T/C44S, genotype 5a, “p7(5a/EUH1480)”) were expressed into inclusion bodies as a fusion to His_9_–trpΔLE using the vector pMM-LR6^64^. The p7 genes (synthetic DNA strings) were ordered from GeneArt (Life Technologies) inserted into vector pMM-LR6 and transformed into XL10 Gold Competent Cells (NEB) for plasmid propagation and storage. The plasmid with the p7 insert was purified from high optical density (600 nm) cultures (QIAprep Spin Miniprep Kit, QIAGEN) and transformed into BL21(DE3) cells (NEB) for protein expression. Genes were confirmed by DNA sequence analysis (Source Bioscience, Oxford). A large culture in LB was grown overnight at 37 °C, followed by condensation into a smaller culture of minimal media the next morning (adapted from^65^). The isotopically labeled p7 peptides were purified via immobilized metal ion affinity chromatography (IMAC) and released from the fusion protein by cyanogen bromide cleavage in 70% formic acid (1 hour, 0.2 g/ml cyanogen bromide). The digest reaction was stopped with 1 N NaOH and dialyzed to water and lyophilized. The dried, cleaved HCV p7(1b/J4) was taken up in 10% SDS, sonicated for 15 minutes, mixed with the same volume of running buffer and filtered through a 0.22 μm membrane (Millex-GS, Millipore) and loaded on a Sephacryl column (HiPrep 26/60 Sephacryl S-200) and separated using an Äkta Pure FPLC system (GE Healthcare)^66^. An isocratic gradient eluted the protein (20 mM sodium phosphate, 1 mM EDTA, 1 mM NaN_3_ and 4 mM SDS, pH 8.2) and the eluate was dialyzed against water and lyophilized^66,67^. The lyophilized, cleaved p7(5a/EUH1480) was purified as previously described^29,30^ using a C18 preparative column (Proto 300 5 μm, Higgins Analytical) on a Gilson PrepLC system (321 Pump, UV/VIS-155 detector, UniPoint software). The pure eluate was freeze-dried.

The lyophilized p7(1b/J4) protein was solubilized directly by addition of Cyclofos-6 (Anatrace, Anagrade) in water at 10× (26.8 mM) or 100× (268 mM) the CMC. The efficiency of solubilization increased with detergent concentration from 26.8 to 268 mM. Insolubilized material was discarded after bench top centrifugation (5 minutes, 16,000 x *g*). Critically, 40 mM sodium phosphate was added after solubilization from a 200 mM stock of pH between 6.0 and 7.0 (as required), and the pH was readjusted between 6.0 and 7.0 with NaOH. Final protein monomeric concentrations were between 200 and 400 μM. All NMR samples contained 5% D_2_O and 0.1 mM 4,4-dimethyl-4-silapentane-1-sulfonic acid (DSS). P7(5a/EUH1480) protein was prepared in a manner similar to that described previously^29^. The lyophilized protein was solubilized in 200 mM DPC (Anatrace, Anagrade) and 6 M guanidine hydrochloride (GuHCl), in the absence of buffer. The protein (~300 μl sample with ~300 μM polypeptide concentration) was refolded upon dialysis against two liters 25 mM MES pH 6.5 in the absence of DPC with two buffer changes at two-hour intervals. Crucially, dialysis was performed using 2 kDa MWCO slide-A-Lyzer 100 µl capacity cups (Thermo Scientific), which provide a surface area that allows full elimination of GuHCl, but retains enough DPC to maintain the protein in solution. The sample was re-equilibrated by dialysis against 30 mL of 200 mM DPC, 25 mM MES pH 6.5 for 24 hours using 10–14 kDa MWCO membranes (Pur-A-Lyzer Mini Dialysis) to allow for detergent exchange. Any insoluble material was removed by centrifugation (5 minutes, 16,000 x *g*). The final protein concentration was ~300 µM.

### NMR spectroscopy and data analysis

All NMR spectra were recorded on NMR spectrometers with Oxford Instrument magnets with ^1^H frequencies between 500 to 950 MHz, equipped with home-built triple resonance probes with triple axis gradients^68^ or with Bruker TCI CryoProbes with single Z-axis gradients (500 and 600 MHz). Experiments were performed at 30 °C or 37 °C as indicated and pH 6.5. NMR spectra were referenced in the direct dimension against DSS at 0 ppm. NMR data were processed using NMRPipe and analyzed using NMRDraw^69^, Analysis^70^ or CARA^71^. The resonances of p7(J4/1b) in Cyclofos-6 could be tentatively assigned using ^15^N-edited NOESY-HSQCs (90 ms and 140 ms mixing times) and a ^15^N-edited TOCSY-HSQC (55 ms mixing time). Assignments were subsequently confirmed using a set of HSQC-based triple-resonance experiments (HNCA, HNCACB/HNCOCACB and HNCO/HNCACO). For characterization of the hydrodynamic properties of the NMR samples, the apparent molecular weights were calculated from the ^15^N R_1_ and R_2_ values by first estimating the rotational correlation time, τ_c_, from the 20% trimmed means of the relaxation rates^72^. Trimmed means were used to exclude residues with internal motions faster or slower than the overall tumbling time. Trimmed means for R_1_ and R_2_ for p7(1b/J4) and p7(5a/EUH1480) were 1.32 s^−1^ and 15.92 s^−1^, and 1.34 s^−1^ and 14.80 s^−1^, respectively. The molecular weight was then calculated from τ_c_ using Stoke’s law^73^ assuming a hydration shell of 1.5 water molecules, a solution viscosity of 0.702 centipoise at 37 °C, and a protein partial specific volume of 0.73 cm^3^/g. To calculate the number of detergent molecules consistent with the scenario of a monomeric p7 embedded in a detergent micelle, the value of the partial specific volume was weighted according to the fraction of the complex that was protein (0.73 cm^3^/g) and detergent (0.94 cm^3^/g).

### Secondary structure calculations

TALOS-N calculations of secondary structure were based on chemical shift data for the following nuclei: ^1^H_N_, ^1^Hα, ^15^N, ^13^C′, and ^13^Cα. Chemical shifts for p7(5a/EUH1480) were obtained from BioMagResBank^74^ entry 19162^29^ and corrected for deuterium isotope effects^75^.

### Molecular dynamics

Structures for MD simulations were immersed in a fully hydrated POPC lipid bilayer of initial dimensions equal to 75 × 75 × 90 Å^3^. Except for the POPC aliphatic tails, which were modeled by means of a united-atom potential energy function^76^, use was made of an all-atom representation. The different components of the assays were described by the macromolecular CHARMM36 (chemistry at Harvard macromolecular mechanics) force field^77^. All the simulations reported herein were performed in the isobaric-isothermal ensemble using the NAMD simulation package^78^. The temperature and the pressure were maintained at 300 K and 1 atm employing, respectively, softly damped Langevin dynamics and the Langevin piston algorithm^79^. Periodic boundary conditions were enforced. The equations of motion were integrated using the r–RESPA multiple-time stepping scheme^80^ with a time step of 2 and 4 fs for short- and long-range interactions, respectively. Non-bonded van der Waals interactions were smoothly switched to zero between 10 and 12 Å. The PME algorithm^81^ was utilized to account for long-range electrostatic interactions. During equilibration of the membrane, soft harmonic restraints were applied to the heavy atoms of the protein, prior to their slow release after appropriate relaxation of the surroundings. Evolution of the three-dimensional structures was monitored over a simulation time of 200 ns. Each production trajectory was prefaced by equilibration of up to 50 ns. For quantitative analysis of conformational changes, the p7 helical structure was decomposed into four regions, wherein TM1 and TM2 were both split into two domains, A (residues 4 to 14), B (residues 20 to 31), C (residues 39 to 47) and D (residues 48 to 54).

## Supplementary information

Supplementary information
