# Supplementary material for: Hepatitis C virus sequence divergence preserves p7 viroporin structural and dynamic features
Source: Sci Rep. 2019 Jun 10;9:8383. doi: 10.1038/s41598-019-44413-x (PMC6557816; doi:10.1038/s41598-019-44413-x)
Supplement: Supplementary file 1 — Supplementary information [file 41598_2019_44413_MOESM1_ESM.pdf]

Hepatitis C virus sequence divergence preserves p7 viroporin structural and dynamic features – Supplementary information

Benjamin P. Oestringer†‡, Juan H. Bolivar†, Jolyon K. Claridge‡§, Latifah Almanea‡, Chris Chipot⌘¶, François Dehez⌘, Nicole Holzmann⌘, Jason R. Schnell‡* & Nicole Zitzmann†*

† Oxford Glycobiology Institute, Department of Biochemistry, University of Oxford, South Parks Road, Oxford, OX1 3QU, United Kingdom.

‡ Department of Biochemistry, University of Oxford, South Parks Road, Oxford OX1 3QU, United Kingdom

⌘ Laboratoire International Associé CNRS-University of Illinois at Urbana Champaign, Université de Lorraine, BP 70239, 54506 Vandœuvre-lès-Nancy, France

¶ Department of Physics, University of Illinois at Urbana-Champaign, 1110 West Green Street, Urbana, Illinois 61801, United States

Corresponding Authors

‡*Department of Biochemistry, University of Oxford, South Parks Road, Oxford OX1 3QU, United Kingdom. Electronic address: Jason.Schnell@bioch.ox.ac.uk

†* Oxford Glycobiology Institute, Department of Biochemistry, University of Oxford, South Parks Road, Oxford, OX1 3QU, United Kingdom. Electronic address: Nicole.Zitzmann@bioch.ox.ac.uk

Present Addresses

†‡ Immunocore Limited, 101 Park Drive, Milton Park, Abingdon, Oxon, OX14 4RY, United Kingdom

‡§ Structural Biology Brussels, Vrije Universiteit Brussel, Pleinlaan 2, 1050 Brussels, Belgium, and Structural and Molecular Microbiology, Structural Biology Research Center, VIB, Pleinlaan 2, 1050 Brussels, Belgium

Supplementary figures

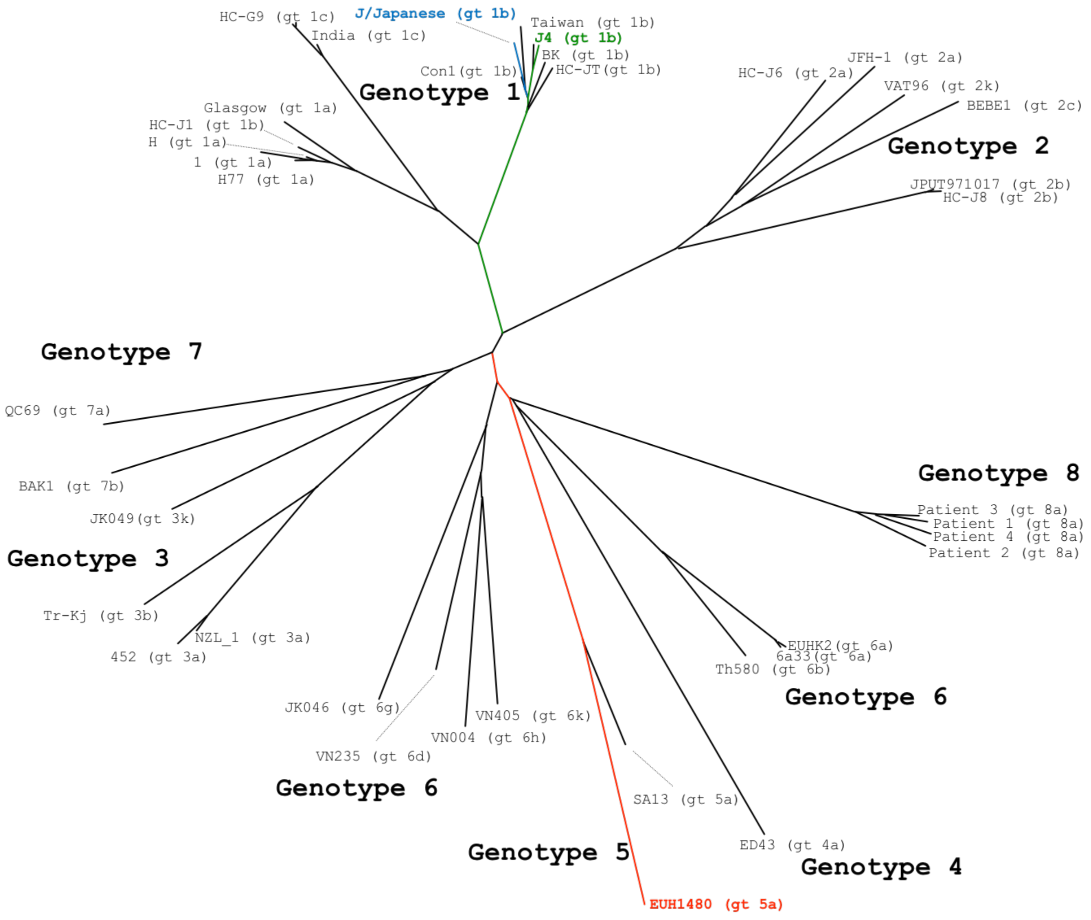

Supplementary Figure 1: Phylogenetic tree of HCV p7 sequences. The isolate name and genotype (gt) are indicated. The tree includes all completely annotated HCV isolates deposited in UniProt. Their accession number can be found in the alignment in **Figure S2**. In addition, isolate BAK1 (gt 7b) and the recently described novel genotype 8 (gt 8a) are included. Genotype 8 has no isolate name assigned and the four identified sequences are listed as patient 1-4.[1-3] Phylogenetic trees including more and different selections of isolates are published elsewhere.[1-5] Isolate J used in Montserret *et al.* is referred to as 'Japanese' in UniProt and J in the tree. Isolate J4 is referred to as (HC-)J4 in UniProt and J4 in the tree. Isolate J, J4 and EUH1480, which are the main isolates studied using NMR (see **Figure 1** of the main text), are highlighted in blue, green and red, respectively. Clustal Omega was used to generate the Newick format tree data and TRex was used to plot the initial radial tree.[6,7]

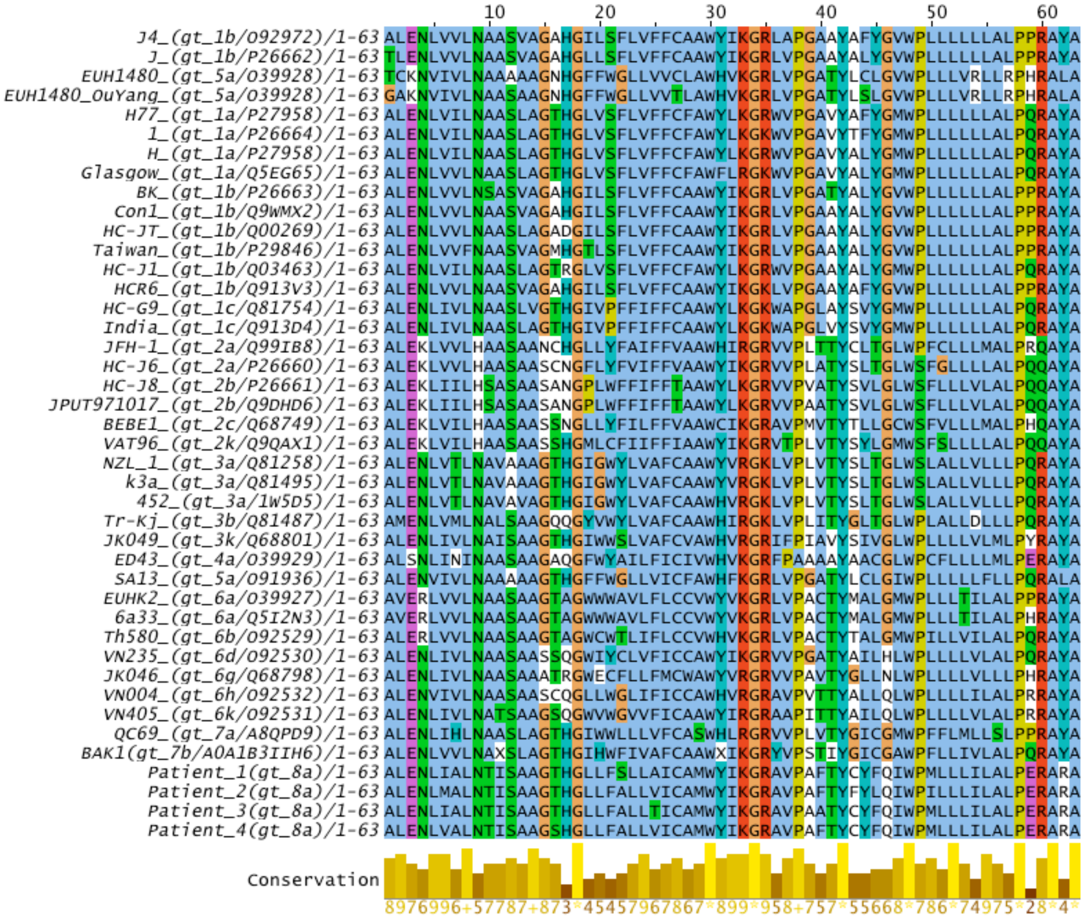

Supplementary Figure 2: Sequence alignment of HCV p7 sequences. The alignment was generated using Jalview.[8] UniProt reviewed HCV p7 sequences including the novel isolate 7b and genotype 8a. Sequence conservation below is shown based on the 41 p7 isolates from genotypes 1a-c, 2a-c, 2k, 3a-b, 3k, 4a, 5a, 6a-b, 6d, 6g-h, 6k and 7a-b (UniProt identification codes/same order as in alignment: O92972, P26662, O39928, P27958, P26664, P27958, Q5EG65, P26663, Q9WMX2, Q00269, P29846, Q03463, Q913V3, Q81754, Q913D4, Q99IB8, P26660, P26661, Q9DHD6, Q68749, Q9QAX1, Q81258, Q81495, Q1W5D5, Q81487, Q68801, O39929, O91936, O39927, Q5I2N3, O92529, O92530, Q68798, O92532, O92531, A8QPD9 and A0A1B3IIH6, respectively). Sequences are colored according to the ClustalX color-scheme, i.e. hydrophobic: blue, positive charge: red, negative charge: magenta, polar: green, cysteines: pink, glycines: orange, prolines: yellow, aromatic: cyan, unconserved: white.[9] Isolate names are shown next to the sequences with the corresponding genotype and UniProt accession number in parentheses where available. At least one example from each of the eight recognized genotypes is included. The four recently identified genotype 8 sequences are listed as patient 1-4.[3] A more complete list of HCV p7 isolates can be found at the International Committee on Taxonomy of Viruses.[10] The isolates of particular interest in this study are J4 p7 (gt 1b, UniProt: O92972) and EUH1480 p7 (gt 5a, UniProt: O39928). The EUH1480 isolate with five mutations (T1G, C2A, A12S, C27T and C44S) used by OuYang *et al.* has also been added.[11] The conservation is depicted as a bar graph below the alignment.

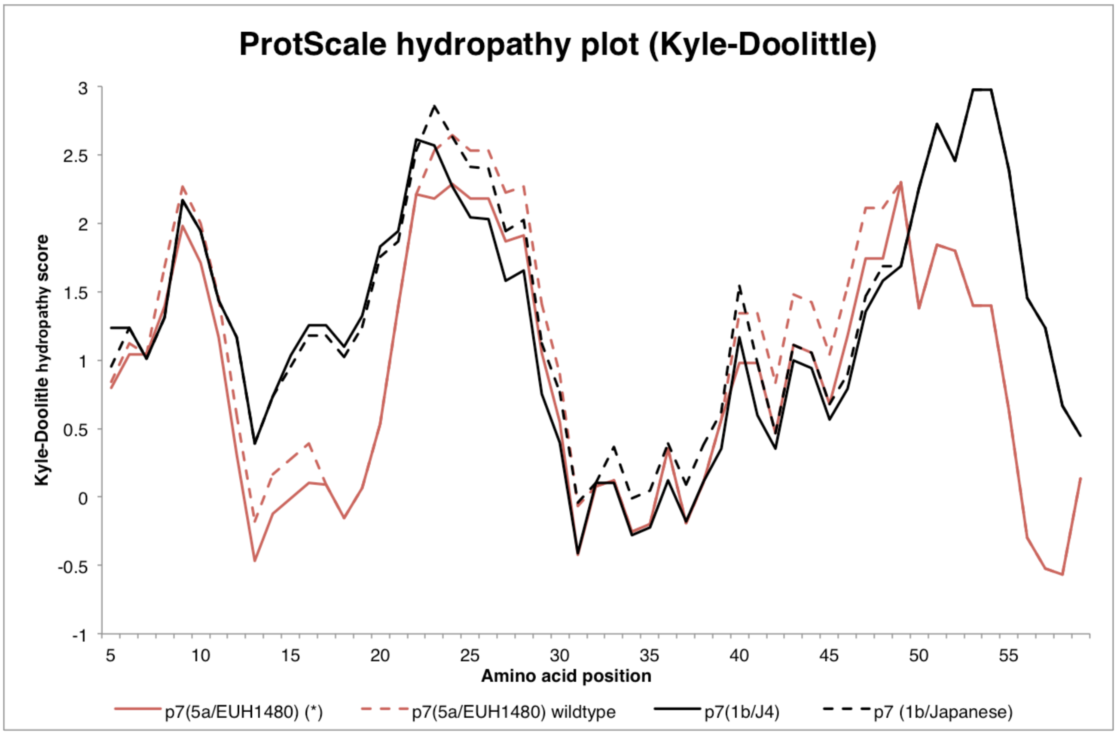

Supplementary Figure 3: The hydropathy index of HCV p7 isolates. The index of p7(5a/EUH1480) used in this study and in OuYang *et al.* with five mutations (*) (T1G, A12S, C2A, C27T, and C44S) and p7(5a/EUH1480) wildtype are plotted in red solid and red dashed lines, respectively. Plots of p7(1b/J4) C27S and p7(1b/Japanese) C27S are shown in black solid and black dashed lines, respectively. The Kyte and Doolittle hydropathy scale with default window size 9 was used. The score is proportional to hydrophobicity.[12,13]

REFERENCES:

1. Smith, D. B. *et al.* Expanded classification of hepatitis C virus into 7 genotypes and 67 subtypes: updated criteria and genotype assignment web resource. *Hepatology* **59,** 318–327 (2014).
2. Salmona, M. *et al.* First next-generation sequencing full-genome characterization of a hepatitis C virus genotype 7 divergent subtype. *Clin. Microbiol. Infect.* **22,** 947.e1–947.e8 (2016).
3. Borgia, S. M. *et al.* Identification of a Novel Hepatitis C Virus Genotype From Punjab, India: Expanding Classification of Hepatitis C Virus Into 8 Genotypes. *J. Infect. Dis.* **218,** 1722–1729 (2018).
4. Bukh, J. The history of hepatitis C virus (HCV): Basic research reveals unique features in phylogeny, evolution and the viral life cycle with new perspectives for epidemic control. *J. Hepatol.* **65,** S2–S21 (2016).
5. Nakano, T., Lau, G. M. G., Lau, G. M. L., Sugiyama, M. & Mizokami, M. An updated analysis of hepatitis C virus genotypes and subtypes based on the complete coding region. *Liver international : official journal of the International Association for the Study of the Liver* **32,** 339–345 (2012).
6. Sievers, F. *et al.* Fast, scalable generation of high-quality protein multiple sequence alignments using Clustal Omega. *Mol. Syst. Biol.* **7,** 539–539 (2011).
7. Boc, A., Diallo, A. B. & Makarenkov, V. T-REX: a web server for inferring, validating and visualizing phylogenetic trees and networks. *Nucleic Acids Research* **40,** W573–9 (2012).
8. Waterhouse, A. M., Procter, J. B., Martin, D. M. A., Clamp, M. & Barton, G. J. Jalview Version 2--a multiple sequence alignment editor and analysis workbench. *Bioinformatics* **25,** 1189–1191 (2009).
9. *jalview clustalX colour schemes*. Available at: http://www.jalview.org/help/html/colourSchemes/clustal.html. (Accessed: 10 December 2018)
10. *International Committee on Taxonomy of Viruses - Confirmed HCV genotypes/subtypes (June 2017)*. Available at: https://talk.ictvonline.org/ictv_wikis/flaviviridae/w/sg_flavi/634/table-1---confirmed-hcv-genotypes-subtypes-june-2017. (Accessed: 10 December 2018)
11. OuYang, B. *et al.* Unusual architecture of the p7 channel from hepatitis C virus. *Nature* **498,** 521–525 (2013).
12. Kyte, J. & Doolittle, R. F. A simple method for displaying the hydropathic character of a protein. *Journal of molecular biology* **157,** 105–132 (1982).
13. Gasteiger, E. *et al.* in *The Proteomics Protocols Handbook* 571–607 (Humana Press, 2005). doi:10.1385/1-59259-890-0:571
